# The triple burden of communicable and non-communicable diseases and injuries on sex differences in life expectancy in Ethiopia

**DOI:** 10.1186/s12939-021-01516-0

**Published:** 2021-08-03

**Authors:** Myunggu Jung, Gizachew Balew Jembere, Young Su Park, William Muhwava, Yeohee Choi, Youngtae Cho, Woorim Ko

**Affiliations:** 1 Faculty of Epidemiology and Population Health, London School of Hygiene & Tropical Medicine, Keppel Street, London, UK; 2 EngenderHealth-Ethiopia, Addis Ababa, Ethiopia; 3 Center for Arts and Humanities, Haverford College, Haverford, PA USA; 4 African Centre for Statistics, United Nations Economic Commission for Africa, Addis Ababa, Ethiopia; 5 Department of Social Welfare, Graduate School of Social Welfare, Ewha Womans University, Seoul, South Korea; 6 Institute of Environment and Health, Population Policy Research Center, Seoul National University, Seoul, South Korea

**Keywords:** Ethiopia, Life table, Decomposition, Life expectancy, Sex differences in life expectancy

## Abstract

**Background:**

Ethiopia has experienced great improvements in life expectancy (LE) at birth over the last three decades. Despite consistent increases in LE for both males and females in Ethiopia, the country has simultaneously witnessed an increasing discrepancy in LE between males and females.

**Methods:**

This study used Pollard’s actuarial method of decomposing LE to compare age- and cause- specific contributions to changes in sex differences in LE between 1995 and 2015 in Ethiopia.

**Results:**

Life expectancy at birth in Ethiopia increased for both males and females from 48.28 years and 50.12 years in 1995 to 65.59 years and 69.11 years in 2015, respectively. However, the sex differences in LE at birth also increased from 1.85 years in 1995 to 3.51 years in 2015. Decomposition analysis shows that the higher male mortality was consistently due to injuries and respiratory infections, which contributed to 1.57 out of 1.85 years in 1995 and 1.62 out of 3.51 years in 2015 of the sex differences in LE. Increased male mortality from non-communicable diseases (NCDs) also contributed to the increased difference in LE between males and females over the period, accounting for 0.21 out of 1.85 years and 1.05 out of 3.51 years in 1995 and 2015, respectively.

**Conclusions:**

While injuries and respiratory infections causing male mortality were the most consistent causes of the sex differences in LE in Ethiopia, morality from NCDs is the main cause of the recent increasing differences in LE between males and females. However, unlike the higher exposure of males to death from injuries due to road traffic injuries or interpersonal violence, to what extent sex differences are caused by the higher male mortality compared to female mortality from respiratory infection diseases is unclear. Similarly, despite Ethiopia’s weak social security system, an explanation for the increased sex differences after the age of 40 years due to either longer female LE or reduced male LE should be further investigated.

**Supplementary Information:**

The online version contains supplementary material available at 10.1186/s12939-021-01516-0.

## Background

Ethiopia has experienced great improvements in life expectancy (LE) at birth over the last three decades. Despite consistent increases in LE for both men and women in Ethiopia, the country has witnessed an increase in sex differences in LE.

Studies on the sex differences in LE are not new. Previous research has found that women generally outlive men in all countries [1] and the sex differences in LE have been widely explained by biological, psychological and social interpretations [2, 3]. Although sex differences in LE are a global phenomenon, they vary according to time and place. Prior studies relate the pattern of sex differences in LE to epidemiological and demographic transition theories. In low-income countries, sex differences in LE tend to be low due to high maternal mortality with high fertility rates [4]. With economic developments and improved living conditions, sex differences in LE start to increase, heralded by significant reductions in fertility and female mortality [5, 6]. Recent studies have also discovered that sex differences in LE have decreased in most high-income countries, as ‘men start to catch up’ [7]. Furthermore, previous studies have demonstrated that the patterns of sex differences in LE along with the epidemiologic transition in high-income countries are recapitulated in low- and middle income countries [3, 7]. The sex differences in LE in many sub-Saharan African countries have remained consistently low in past decades, generally decreasing until around 2000, but have stared increasing in recent times, creating a U-shaped curve [8]. It is important to explore overall trends of sex differences in LE but it is also essential to pay close attention to the roles of specific causes and ages of death in patterns of sex differences in LE over time [9]. One way to enhance our understanding of the patterns of sex differences in LE is to decompose LE by age- and cause- specific mortality. Although several studies on sex differences in LE in high- and middle- income countries have decomposed the contribution of age and cause to changes in sex differences in LE [10, 11], there are few studies in sub-Saharan African countries largely due to the lack of official statistics on ages and causes of death [12]. In addition, previous age- and cause- specific decomposition studies on sex differences in LE in high- and middle- income countries have investigated the ∩ − shaped patterns of sex differences in LE [9–11, 13], because the ∩ − shape pattern of sex differences in LE has been observed in most middle-income countries over the past three decades and the sex differences in LE have been continuously decreasing in high-income countries. However, during the same period, sub-Saharan African countries have witnessed the opposite pattern of sex differences in LE (U-shape). Despite a few existing studies on age- and cause- specific contributions to sex differences in LE in sub-Saharan African countries, their age- and cause- decomposition analyses have only focused on relatively short periods [14], which are unable to explain the role of age and cause specific mortality in the U-shaped patterns of sex differences in LE over the decades. To the best of our knowledge, this is the first paper to investigate age- and cause- specific contributions to changes in sex differences in LE of a sub-Saharan African country over several decades. Therefore, this research contributes to the literature on temporal trends of sex differences in LE in sub-Saharan Africa by assessing a more comprehensive list of age- and cause- specific mortality. Among sub-Saharan African countries, Ethiopia is a good case study as the increase in sex differences in LE has been one of the highest among sub-Saharan African countries over the past three decades, although, according to the Global Burdens of Disease (GBD) study by the Institute for Health Metrics and Evaluation (IHME), Ethiopia’s sex differences in LE are still below the world average; in 2015, the difference in LE between males and females was 3.51 years for Ethiopia and 4.26 years for the world. While Ethiopia has enjoyed consistent improvements in LE since the 1990s, the country has also experienced an epidemiological transition from high mortality due to communicable diseases, mainly affecting young people, to increasing mortality due to non-communicable diseases (NCDs), affecting mainly middle-aged and older people [15, 16]. It is widely known that male and female epidemiological patterns are not analogous, but radically different [17]. Moreover, growing civil unrest in the country following political changes and COVID-19 have the potential to result in a disproportionate increase in the difference in mortality between men and women [18–20]. Hence, identifying sex-, age- and cause- specific LE differences can provide an in-depth insight into why sex differences in LE have changed over and what to be prepared for in response to imminent public health threats, such as civil unrest and Ethiopia’s high road traffic accidents in pursuit of improved health outcomes, including sustainable improvements in LE for both men and women.

The overarching goal of this paper is to investigate and quantify the age- and cause- specific drivers of the pattern of sex differences in LE in Ethiopia between 1995 and 2015 in five-year intervals. First, we compare the contributions of age and cause to the improvements in male and female life expectancy between 1995 and 2015. Then, we investigate the underlying age- and cause- specific contributions to changes in sex differences in LE in Ethiopia between 1995 and 2015. We do this by analysing the impact of 1) changes in age- and cause- specific mortality on the increases in LE in men and women; 2) changes in age- and cause- specific mortality on sex differences in LE.

## Methods

### Data

**Fig. 1:**
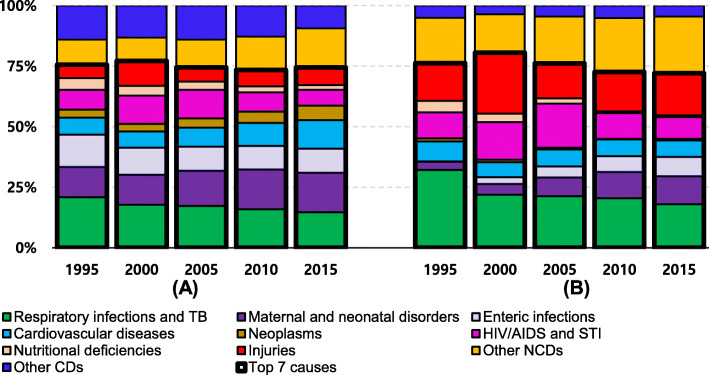
Proportion of top seven causes of death in Ethiopia between 1995 and 2015 in five-year intervals

This study obtained data of Ethiopia’s LE and age- and cause- specific deaths from the Global Burden of Disease (GBD) 2017 study [16]. The GBD data is publicly available on the Global Health Data Exchange website (http:// ghdx.healthdata.org). The GBD 2017 study follows the Guidelines for Accurate and Transparent Health Estimates Reporting (GATHER), which includes recommendations on documentation of data sources, estimation methods, and statistical analysis. Detailed methods for the GBD 2017 study are provided in other publications [16]. The GBD 2017 study organises causes of death into a hierarchical list consisting of four levels. At the highest level (Level 1), all disease burdens are divided into three mutually exclusive and collectively exhaustive categories: communicable, maternal, neonatal, and nutritional diseases (CMNNDs); non-communicable diseases (NCDs); injuries. Level 2 distinguishes the Level 1 category into 22 cause groups. Among the 22 causes of death, the top 7 causes of death were filtered out, and the remaining causes were merged into ‘other communicable diseases (CDs)’, ‘other non-communicable diseases (NCDs)’ and ‘injuries’ in accordance with IHME’s categories. Due to diversity in data sources and sparsity of data for many low-income countries, the GBD 2017 data is available in the form of the number of deaths in point estimates and 95% uncertainty intervals. Figure 1 shows the final 10 causes of death for this study. Figure 1 (A) shows the proportions of each cause in the total number of deaths and Fig. 1 (B) illustrates the proportions of cause-specific differences in the number of deaths between males and females. Although the proportions of each cause of death in the top seven causes changed over time, the total proportion of the top seven causes and injuries represented by thicker box lines in Fig. 1 remained similar at around 75% of both the total number of deaths and the total differences in the number of deaths between males and female over the 20 years.

### Analysis

In order to compare cause-specific mortality rates between male and female over time, the age-standardised mortality rates (ASMR) was used to quantify the trends associated with mortality in the 10 causes for both males and females between 1995 and 2015 in five-year intervals []. The ASMR (per 100,000 population) was calculated according to the direct method by summing up the products of age-specific rates (*a*_*i*_, where *i* denotes the *i* th age group) and the number of persons (*P*_*i*_) in the same age subgroup *i* of the chosen reference standard population, divided by the sum of the standard population weights, i.e., [11].

$$ ASMR=\frac{\sum_{i=1}^{Age\  groups}{a}_i{P}_i}{\sum_{i=1}^{Age\  groups}{P}_i}\times \mathrm{100,000} $$

Standardisation can eliminate the influence of internal differences (such as sex, age, etc.) in the male and female groups, and allows the analysis of any substantive differences. The population to be used as a reference was derived from the average of the age distributions of the male and female population for the five study years at each age group. In order to address the uncertainty of the GBD 2017 data, 95% uncertainty intervals were used to show uncertainty ranges of cause-specific ASMRs for males and female. The age-standardised rate ratio can be calculated by dividing the age-adjusted rate for a population group by that of the referenced population group. This relative measure is called the comparative mortality figure (CMF) [21]. The CMF was used to compare ASMRs between males and females.

The Arriaga and Pollard methods are commonly used for age-cause decompositions of mortality differences [22, 23]. Previous studies have shown that results from the two methods are quite similar when appropriate formulae are applied to a set of data [24]. In addition, studies indicate that the Pollard method is an exact decomposition developed using a continuous modelling approach of the life table, while the Arriaga method is an approximate method based on the discrete analysis approach [24]. Therefore, this study uses the Pollard method of decomposing LE to quantify the age- and cause- specific contributions to changes in LE and sex differences in LE [20, 21]. The Pollard method allows for decomposing both different age groups and different causes of death.

$$ {e}_0^2-{e}_0^1=\sum \limits_{i=1}^n\sum \limits_{x=0}^k\left({iQ}_x^1-{iQ}_x^2\right)\ast {W}_x $$

where, $$ {W}_x=\frac{1}{2}\ \left({xP}_0^2{e}_x^1+{xP}_0^1{e}_x^2\right) $$

where, ${e}_0^1$ and ${e}_0^2$ are the LE at birth for specific times in 1 and 2, *n* denotes the number of considered causes of death (10 causes for this study), and *k* the last included age intervals. ${iQ}_x^1$ is the mortality rates of the *i-th* cause of death at age interval *x* with the weight *W*_*x*_. ${xP}_0^1$ and ${xP}_0^2$ denote the probability of living from birth (0) to age *x* (0+ *x*) at time points 1 and 2. Since mortality rates in the GBD 2017 database were reported for five-year age groups, the equation had to be adapted accordingly. The age-specific change in LE was reported in five categories: 0–4, 5–14, 15–39, 40–64 and ≥ 65 years. The age- and cause-specific change in LE and sex differences in LE were calculated for adjacent time intervals of five-years starting in 1995 and ending in 2015 (1995–2000, 2000–2005, 2005–2010, 2010–2015). For sex differences in LE, a similar method was used;

$$ {e}_0^f-{e}_0^m=\sum \limits_{i=1}^n\sum \limits_{x=0}^k\left({iQ}_x^m-{iQ}_x^f\right)\ast {W}_x $$

where, $$ {W}_x=\frac{1}{2}\ \left({xP}_0^f{e}_x^m+{xP}_0^m{e}_x^f\right) $$

where, ${e}_0^f$ and ${e}_0^m$ are the LE at birth for females(f) and males(m) 2, *n* denotes the number of considered causes of death (10 causes for this study), and *k* the last included age intervals. ${xP}_0^f$ and ${xP}_0^m$ denote the probability of living from birth (0) to age *x* (0+ *x*) for females(f) and males(m). This study used the mean values of the number of deaths from the GBD estimates for the age- and cause- specific decomposition analysis. Although previous studies used relative measures, such as the male-to-female ratio of life expectancy at birth, to explore the trends of differences in LE between males and female, we only focused on absolute differences to decompose age- and cause- specific contributions to changes and differences in LE between males and females [7].

## Results

### Trends of life expectancy and age -standardised mortality rates in Ethiopia

**Fig. 2:**
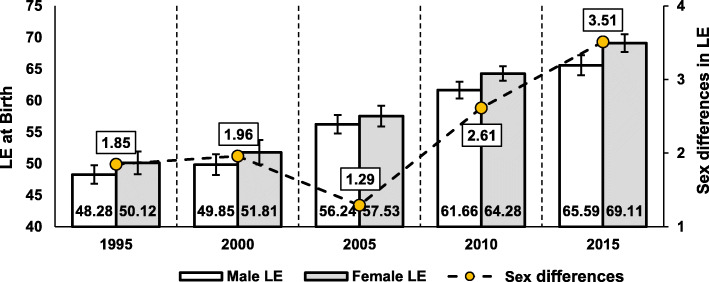
Trends in male and female LE and sex differences in LE for Ethiopia, 1995–2015

Figure 2 shows changes in LE with 95% uncertainty ranges for both males and females and the sex differences in LE between 1995 and 2015 in five-year intervals. The figure shows that LE for both men and women steadily increased with female LE always higher than male LE. However, the sex differences in LE were low (1.85 years) in 1995 and slightly increased to 1.95 years in 2000. After a drop to 1.29 years in 2005, the sex differences in LE steadily increased to 2.61 years and 3.51 years in 2010 and 2015, respectively. Table 1 shows the age-standardised mortality rates (ASMRs) per 100,000 population of the 10 causes of death for both males and females with 95% uncertainty ranges. The CMF in Table 1 indicates the ratio of the standardised mortality rate to the crude death rate in a standard population. The CMF shows that sex differences in age-standardised all-cause mortality had similar patterns to the patterns in sex differences in LE. All-cause mortality for males was 20% higher than for females in 1995. The figure then increased to 21% in 2000, decreased to 19% in 2005 and then increased again to 28 and 31% in 2010 and 2015, respectively. The standardised all-cause mortality increased from 1068.9 to 1093.0 per 100,000 persons for females and from 1287.0 to 1316.9 for males between 1995 and 2000, which was mainly attributed to increased deaths from injuries since the ASMR from both CMNNDs and NCDs declined during the same period. Although the increase in deaths by injuries during 1995–2000 is further discussed below, it should be noted that the exceptional increase in ASMR from injuries from 1995 to 2000 was a direct consequence of the Eritrean-Ethiopian war between 1998 and 2000. After the war, between 2000 and 2015, age-standardised all-cause mortality steadily decreased to 646.1 for females and 846.1 for males in 2015. Although the ASMR from NCDs increased from 202.2 to 215.7 for females and from 272.3 to 291.2 for males between 2000 and 2015, the increased figure due to NCDs was offset by a decline in ASMR from both CMNNDs and injuries. While the ASMR from most causes was higher for males than for females over the study periods, the female ASMR from HIV/STIs and neoplasms was always higher than for males

**Table 1:** Age-standardised mortality rate per 100,000 population for Ethiopia from 1995 to 201.

Cause of death	1995		2000		2005		2010		2015	
	Female	Male	Female	Male	Female	Male	Female	Male	Female	Male
**Communicable, Maternal, Neonatal and Nutritional diseases**	**819.1 (577.6–1151.9)**	**918.8 (673.0–1257.0)**	**809.7 (575.5–1107.0)**	**878.8 (655.6–1160.6)**	**683.1 (510.4–904.0)**	**746.9 (575.2–952.8)**	**501.3 (386.4–654.8)**	**586.6 (458.5–748.9)**	**397.6 (302.6–529.0)**	**479.7 (365.6–618.6)**
Nutritional deficiencies	50.1 (27.2–83.7)	63.7 (39.3–90.0)	42.3 (23.3–68.8)	54.0 (37.6–71.6)	30.1 (20.3–43.0)	36.4 (26.0–46.0)	19.6 (15.3–25.0)	21.5 (16.9–27.0)	14.7 (10.7–20.3)	14.4 (11.2–18.2)
Respiratory infections and tuberculosis	199.6 (163.7–236.1)	291.8 (242.3–344.7)	177.9 (148.5–208.9)	251.4 (215.9–285.5)	143.1 (121.5–169.0)	204.7 (181.7–229.4)	105.9 (92.4–121.4)	158.9 (141.9–177.5)	86.9 (73.2–101.3)	130.8 (112.6–149.3)
Maternal and neonatal disorders	142.6 (127.0–161.5)	152.6 (129.3–178.6)	140.7 (124.9–158.6)	155.6 (133.7–179.8)	135.7 (119.9–153.2)	158.1 (132.0–186.5)	122.7 (105.0–143.0)	150.7 (120.8–184.2)	107.6 (91.5–127.3)	136.0 (106.9–169.8)
HIV/AIDS and STI	116.0 (70.7–173.2)	81.2 (50.9–122.8)	166.8 (111.0–235.4)	114.5 (76.7–159.6)	145.9 (107.6–188.2)	92.9 (68.4–121.2)	80.2 (65.2–99.0)	52.7 (42.0–67.1)	59.1 (46.9–73.5)	38.6 (30.2–49.6)
Enteric infections	156.6 (89.6–246.9)	157.0 (102.9–250.2)	129.8 (78.1–196.5)	139.3 (95.1–206.8)	93.8 (63.5–137.2)	107.0 (78.5–144.1)	72.9 (48.8–106.4)	89.7 (66.8–117.2)	65.3 (41.9–103.3)	84.7 (57.8–113.8)
Other CDs	158.2 (99.3–250.4)	172.6 (108.3–270.7)	152.2 (89.7–238.7)	164.1 (99.7–256.3)	134.6 (77.6–213.5)	147.8 (88.5–225.7)	99.9 (59.7–160.0)	113.1 (70.0–175.9)	64.0 (38.4–103.2)	75.1 (46.9–117.8)
**Non-communicable diseases**	**206.0 (137.8–285.8)**	**279.5 (194.8–379.9)**	**202.2 (145.4–270.6)**	**272.3 (204.0–357.4)**	**196.7 (151.0–256.4)**	**270.4 (210.0–347.3)**	**195.5 (159.6–243.3)**	**270.5 (221.1–335.8)**	**215.7 (170.7–272.5)**	**291.2 (234.2–365.3)**
Neoplasms	40.1 (28.1–51.4)	36.7 (25.5–50.7)	39.5 (31.0–49.6)	36.1 (26.4–47.4)	39.1 (32.4–47.7)	37.2 (28.7–46.6)	39.5 (33.1–46.8)	39.0 (30.6–46.5)	44.8 (36.1–54.2)	43.6 (34.5–52.1)
Cardiovascular diseases	71.0 (51.5–90.5)	94.8 (71.9–114.5)	71.0 (53.9–87.2)	91.8 (75.8–106.3)	68.8 (56.5–81.5)	89.1 (76.0–101.8)	69.8 (61.3–77.3)	88.0 (78.7–99.0)	79.9 (67.5–91.4)	95.6 (82.0–110.9)
Other NCDs	94.8 (58.3–143.9)	148.0 (97.4–214.7)	91.7 (60.4–133.8)	144.4 (101.8–203.7)	88.7 (62.1–127.2)	144.1 (105.4–198.9)	86.2 (65.2–77.3)	143.5 (111.9–190.2)	91.0 (67.1–126.9)	151.9 (117.6–202.3)
**Injuries**	43.8 (34.5–54.9)	88.7 (70.3–106.8)	81.1 (63.6–126.6)	165.8 (131.0–259.5)	38.5 (31.5–46.6)	80.3 (67.0–99.0)	35.2 (29.0–42.6)	77.3 (63.7–95.5)	32.8 (26.4–40.8)	75.2 (60.9–94.7)
**All causes**	**1068.9 (749.9–1492.6)**	**1287.0 (938.1–1743.8)**	**1093.0 (784.5–1504.2)**	**1316.9 (990.6–1777.5)**	**918.2 (692.9–1207.1)**	**1097.7 (852.2–1399.1)**	**732.0 (575.0–940.7)**	**934.4 (743.3–1180.2)**	**646.1 (499.7–842.3)**	**846.1 (660.7–1078.5)**
**CMF**	**1.00**	**1.20**	**1.00**	**1.21**	**1.00**	**1.19**	**1.00**	**1.28**	**1.00**	**1.31**

### Age- and cause- specific contributions to changes in LE for both males and females

**Fig. 3:**
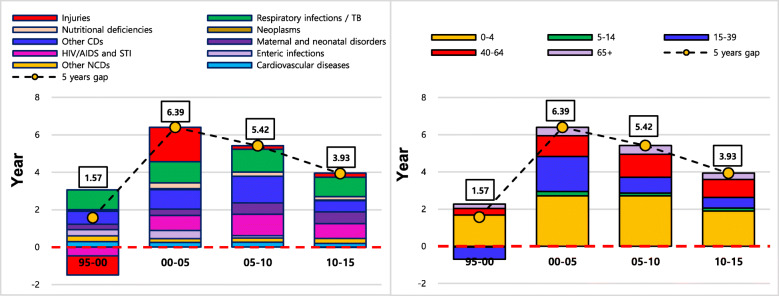
Five-year contribution of age- and cause- specific mortality to changes in male LE

**Fig. 4:**
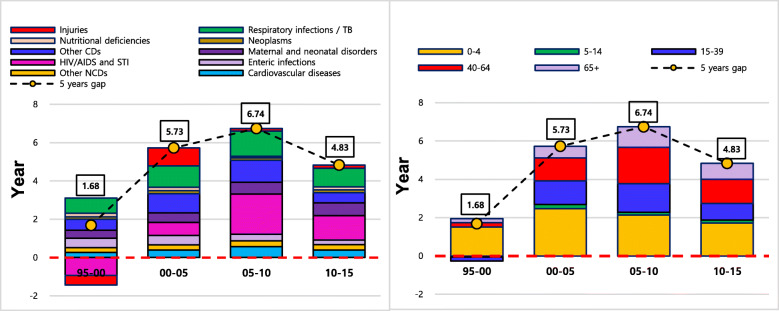
Five -year contribution of age- and cause- specific mortality to changes in female LE

Tables 2 and 3 represent the numerical contributions of age- and cause- specific mortality to changes in LE for males and females in five-year intervals between 1995 and 2015, where negative figures indicate decreasing effects on LE. In terms of cause-specific contributions, CMNNDs were the main causes of increases in LE for both males and females over the study periods, contributing to 1.53 out of 1.68 years during 1995-2000 and 3.84 out of 4.83 years during 2010-2015 of the increases in male LE and 1.91 out of 1.57 years during 1995-2000 and 3.20 out of 3.93 years induring 2010-2015 of the increases in female LE. Although the decreasing effects of injuries and HIV/AIDS and STIs on both male and female LE were observed between 1995 and 2000, the decreasing effect of injuries were particularly stronger on female LE. In terms of age-specific contributions to changes in LE for males and females, the contributions of the 0–4 age group to both male and female LE were the main causes of the increases over the study periods. Figures 3 and 4 illustrate the contributions of age- and cause- specific mortality to LE at birth for both males and females in five-year intervals between 1995 and 2015, where bars below the x-axis at zero indicate decreasing effects on LE between corresponding study periods. In terms of cause- specific contributions, reduced mortality from CMNNDs such as respiratory infections and TB, maternal and neonatal disorders, HIV/AIDS and STIs, and other CDs, explained most of the increases in LE for both males and females. Among the 10 causes of death over all the study periods, injuries and HIV/AIDS and STIs only had negative effects on life expectancy gains for both males and females between 1995 and 2000. The 10 causes of death contributed positively (indicating a reduction in burden of disease) to LE improvement over the rest of the study periods after 2000. Between 2000 and 2005, improvements in LE were largely attributed to decreased mortality from injuries for both males and females, particularly for males; after that, the positive effects of injuries on LE became marginal. Reduced female mortality in HIV/AIDs and STIs contributed to the larger increments in female LE between 2005 and 2015. In terms of age- specific contributions, apart from the period 1995–2000, LE gains were observed in all age groups over the study periods. Between 1995 and 2000, negative effects of the 15–39 age group on LE gains were identified for both males and females, but mainly for males due to the greater male involvement in the Eritrean-Ethiopian war. The contribution of the 0–4 age group to the increases in LE was apparent for both males and females. Since 2000, despite the consistent contribution of the 0–4 age group to improvements in LE for both males and females, other age groups increasingly contributed to increases in LE. In comparison to age-specific contributions to male LE, the larger contributions of the 15–39 and 65+ female age groups to female LE from 2000 to 2015 were notable.

**Table 2:** Age-specific contributions to changes in LE for males and females in Ethiopia

Age	Male, Year (%)				Female, Year (%)			
	1995–2000	2000–2005	2005–2010	2010–2015	1995–2000	2000–2005	2005–2010	2010–2015
0–4	1.51 (89.8)	2.47 (43.1)	2.13 (31.6)	1.72 (35.6)	1.68 (106.8)	2.71 (42.4)	2.72 (50.2)	1.89 (48.0)
5–14	−0.06(−3.9)	0.22 (3.8)	0.15 (2.3)	0.15 (3.1)	−0.04(−2.5)	0.24 (3.7)	0.15 (2.7)	0.16 (4.0)
15–39	−0.20(−11.8)	1.23 (21.5)	1.50 (22.2)	0.88 (18.1)	−0.66(−41.9)	1.88 (29.4)	0.84 (15.5)	0.58 (14.8)
40–64	0.23 (13.4)	1.20 (21.0)	1.90 (28.1)	1.26 (26.1)	0.35 (22.3)	1.12 (17.6)	1.24 (22.8)	0.97 (24.6)
65+	0.21 (12.4)	0.60 (10.5)	1.06 (15.8)	0.82 (17.0)	0.24 (15.3)	0.44 (6.9)	0.48 (8.8)	0.34 (8.6)
**Total**	**1.68 (100)**	**5.73 (100)**	**6.74 (100)**	**4.83 (100)**	**1.57 (100)**	**6.39 (100)**	**5.42 (100)**	**3.93 (100)**

Note: Results in Year refer to age-specific contributions to the total years of changes in life expectancy (LE) in the last row. Results in % are derived from dividing the total years of changes in LE by contributions of each age group.

**Table 3:** Cause-specific contributions to changes in LE for males and females in Ethiopia

Cause of death	Male, Year (%)				Female, Year (%)			
	1995–2000	2000–2005	2005–2010	2010–2015	1995–2000	2000–2005	2005–2010	2010–2015
**Communicable, Maternal, Neonatal and Nutritional diseases**	**1.53 (90.7)**	**3.96 (69.1)**	**5.60 (83.1)**	**3.84 (79.6)**	**1.91 (121.3)**	**4.05 (63.3)**	**4.73 (87.3)**	**3.20 (81.5)**
Respiratory infections and TB	0.80 (47.8)	1.12 (19.5)	1.31 (19.4)	0.97 (20.1)	1.06 (67.5)	1.12 (17.6)	1.21 (22.4)	1.03 (26.1)
Enteric infections	0.49 (29.0)	0.49 (8.5)	0.34 (5.0)	0.25 (5.1)	0.33 (21.0)	0.44 (6.8)	0.13 (2.4)	−0.02 (−0.5)
Maternal and neonatal disorders	0.43 (25.3)	0.51 (8.8)	0.61 (9.1)	0.68 (14.1)	0.29 (18.4)	0.35 (5.5)	0.61 (11.2)	0.63 (15.9)
HIV/AIDS and STI	−0.93 (−55.1)	0.67 (11.7)	2.10 (31.2)	1.26 (26.0)	−0.46 (−29.2)	0.80 (12.5)	1.14 (21.0)	0.79 (20.2)
Nutritional deficiencies	0.18 (10.5)	0.18 (3.1)	0.10 (1.4)	0.16 (3.2)	0.00 (−0.3)	0.32 (5.0)	0.22 (4.1)	0.18 (4.5)
Other CDs	0.56 (33.2)	1.00 (17.4)	1.15 (17.0)	0.53 (11.0)	0.69 (43.8)	1.02 (16.0)	1.42 (26.1)	0.60 (15.3)
**Non-communicable diseases**	**0.66 (39.2)**	**0.83 (14.5)**	**1.00 (14.8)**	**0.82 (16.9)**	**0.69 (43.9)**	**0.51 (8.0)**	**0.49 (9.1)**	**0.50 (12.8)**
Cardiovascular diseases	0.26 (15.5)	0.40 (7.0)	0.57 (8.4)	0.40 (8.3)	0.29 (18.5)	0.25 (3.9)	0.27 (4.9)	0.20 (5.1)
Neoplasms	0.14 (8.2)	0.16 (2.7)	0.12 (1.7)	0.14 (2.9)	0.08 (5.4)	0.06 (0.9)	0.01 (0.2)	0.04 (1.0)
Other NCDs	0.26 (15.5)	0.27 (4.8)	0.31 (4.6)	0.28 (5.8)	0.31 (20.0)	0.20 (3.2)	0.22 (4.0)	0.27 (6.8)
**Injuries**	**−0.50 (−29.9)**	**0.94 (16.4)**	**0.14 (2.1)**	**0.17 (3.6)**	**−1.03 (−65.3)**	**1.83 (28.6)**	**0.19 (3.6)**	**0.22 (5.7)**
**Total**	**1.68 (100)**	**5.73 (100)**	**6.74 (100)**	**4.83 (100)**	**1.57 (100)**	**6.39 (100)**	**5.42 (100)**	**3.93 (100)**

Note: Results in Year refer to cause-specific contributions to the total years of changes in life expectancy (LE) in the last row. Results in % are derived from dividing the total years of changes in LE by contributions of each cause.

### Age- and cause- specific contributions to sex differences in LE

**Fig. 5:**
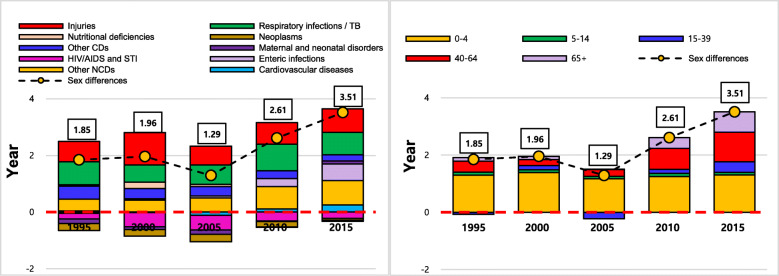
Sex differences in LE by age and cause for males and females

Figure 5 shows the contributions of age- and cause- specific mortality to the sex differences in LE for each study year between 1995 and 2015, where bars below the x-axis at zero indicate decreasing effects on sex differences in LE due to higher female mortality. Tables 4 and 5 represent the numerical contributions of age- and cause- specific mortality to the sex differences in LE, where negative figures also indicate decreasing effects on the sex differences in LE due to higher female mortality. In terms of cause-specific contributions, injuries and respiratory infections were two the main causes of sex differences in LE over the study period, contributing to 1.57 out of 1.85 years in1995 and 1.62 out of 3.51 years in 2015 of the sex differences in LE.

**Table 4:** Age-specific contributions to sex differences in LE in Ethiopia

Age	1995		2000		2005		2010		2015	
	Year	%	Year	%	Year	%	Year	%	Year	%
0–4	1.29	70.1	1.39	71.1	1.26	90.6	1.26	48.1	1.31	37.2
5–14	0.11	6.2	0.09	4.7	0.10	7.0	0.11	4.0	0.09	2.6
15–39	− 0.07	−3.6	0.14	7.3	−0.32	−17.1	0.14	5.2	0.37	10.5
40–64	0.38	20.6	0.21	10.9	0.24	17.8	0.74	28.2	1.03	29.4
65+	0.12	6.7	0.12	6.1	0.02	1.6	0.38	14.5	0.71	20.2
**Total**	**1.85**	**100**	**1.96**	**100**	**1.29**	**100**	**2.61**	**100**	**3.51**	**100**

Note: Results in Year refer to age-specific contributions to the total years of sex differences in life expectancy (LE) in the last row. Results in % are derived from dividing the total years of differences in LE by contributions of each age group.

**Table 5:** Cause-specific contributions to sex differences in LE in Ethiopia

Cause of death	1995		2000		2005		2010		2015	
	Year	%	Year	%	Year	%	Year	%	Year	%
**Communicable, Maternal, Neonatal and Nutritional diseases**	**0.92**	**49.9**	**0.65**	**33.1**	**0.50**	**38.6**	**1.14**	**43.7**	**1.624**	**46.33**
Respiratory infections and TB	0.82	44.3	0.60	30.8	0.68	52.8	0.94	35.9	0.79	22.6
Enteric infections	−0.04	−2.2	0.05	2.6	0.07	5.4	0.28	10.8	0.659	19.77.6
Maternal and neonatal disorders	−0.16	−8.6	− 0.09	−4.8	− 0.15	−11.8	− 0.02	−0.7	0.11	3.1
HIV/AIDS and STI	−0.20	− 10.8	− 0.50	−25.6	− 0.51	−39.6	−0.31	− 12.0	− 0.22	−6.36
Nutritional deficiencies	0.05	2.6	0.23	11.7	0.08	6.2	−0.02	−0.8	− 0.04	−1.1
Other CDs	0.45	24.6	0.36	18.3	0.33	25.6	0.28	10.6	0.29	8.3.3
**Non-communicable diseases**	**0.21**	**11.3**	**0.17**	**8.5**	**0.12**	**9.3**	**0.71**	**27.2**	**1.05**	**30.0**
Cardiovascular diseases	0.04	2.1	−0.02	−1.1	−0.12	−9.0	0.11	4.4	0.25	7.1
Neoplasms	−0.25	−13.4	−0.23	−11.9	− 0.26	−20.5	−0.19	−7.2	− 0.06	− 1.7
Other NCDs	0.42	22.6	0.42	21.5	0.50	38.8	0.78	30.0	0.86	24.6
**Injuries**	**0.72**	**38.8**	**1.14**	**58.3**	**0.67**	**52.1**	**0.76**	**29.1**	**0.83**	**23.7**
**Total**	**1.85**	**100**	**1.96**	**100**	**1.29**	**100**	**2.61**	**100**	**3.51**	**100**

Note: Results in Year refer to cause-specific contributions to the total years of differences in LE between males and females in the last row. Results in % are derived from dividing the total years of differences in LE by contributions of each cause.

The increase in sex differences in LE between 1995 and 2000 was notably attributed to higher mortality due to injuries in men. Although decreasing effects of HIV/AIDS and STIs on sex differences in LE from − 0.20 years in 1995 to − 0.50 years in 2000 were observed, the decreasing effects were offset by stronger effects of injuries from 0.72 years in 1995 to 1.14 years in 2000. Between 2000 and 2005, the strong effect by HIV/AIDS and STIs on sex differences in LE (− 0.50 years and − 0.51 years) combined with diminished contributions of injuries to sex differences in LE (1.14 years and 0.67 years) resulted in a decrease in the overall sex differences in LE between 2000 and 2005. Between 2005 and 2015, gradually increasing contributions of NCDs, including cardiovascular diseases (0.12 years to 1.05 years), coupled with a reduction in decreasing effects of HIV/AIDS and STIs (− 0.51 years to − 0.22 years) led to a steady increase in sex differences in LE. In terms of age- specific contributions to sex differences in LE, the contribution of the 0–4 age group to sex differences in LE was strong between 1995 and 2005. However, sex differences in LE were increasingly observed in the 40–64 age group and older age groups in 2010 and 2015. The increased contribution of the elderly group to the sex differences in LE is mainly attributed to the reduction in age specific mortality among females in this age group.

## Discussion

Although previous studies have explored the trends of sex differences in LE in sub-Saharan Africa, the impact of age- and cause- specific mortality on temporal patterns of sex differences in LE in sub-Saharan Africa is as yet almost unknown. Therefore, this study attempted to investigate age- and cause- specific mortality contributions to sex differences in LE in Ethiopia between 1995 and 2015 in five-year intervals. First, we compared age- and cause- specific contributions to the improvements in male versus female life expectancy. Then, we investigated the underlying age- and cause- specific contributions to sex differences in LE in Ethiopia.

With regard to increments in LE for males and females, both males and females experienced a similar pattern of increases in LE at birth in all age groups and all the selected causes of death, except during the period where Ethiopia was at war with Eritrea. For both males and females, reductions in mortality from CDs such as respiratory infections, TB and ‘other CDs’ explained many of the increases in LE during the study periods. The epidemiological transition theory provides the theoretical background for the improvement in LE for both males and females in Ethiopia between 1995 and 2015. Our results from ASMRs and decomposition analysis show that reductions in deaths from the 0–4 age group profoundly contributed to the improvement in LE, and the ASMR from CMMNDs was gradually overtaken by the ASMR from NCDs for both males and females in Ethiopia.

Despite the analogous patterns, differences between male and female improvements in LE were also identified. First, although injuries and HIV/AIDS and STIs were the two main factors responsible for decreasing LE for both males and females during the war-affected period (1995–2000), the biggest decreasing effect was noted from injuries for males and HIV/AIDS/STIs for females. This result is consistent with previous studies that show that men are more directly affected by wars, while women are more indirectly affected by wars due to, for instance, inadequate reproductive health care systems or gender-based violence in war time [25–27]. Second, while increases in female LE were higher than that of males during most study periods, male improvements in LE (6.39) were greater than that of females (5.73) between 2000 and 2005. The exceptionally large contributions to male LE by injuries between 2000 and 2005 compared to other study periods suggests that it was largely the consequence of male mortality reduction due to injuries after the war. Furthermore, since 2005 female deaths from diseases related to sexual reproductive health declined more than male death, which coincides with prior studies that show that socioeconomic developments along with public health improvements reduce maternal mortality and fertility rates, and thereby increasing women’s health status. It should be noted that the Ethiopian government in 2003 launched its flagship health service delivery system, the Health Extension Programme (HEP), to increase access to and use of sexual and reproductive health care [28, 29]. Previously, the contraceptive prevalence rate (CPR) for modern methods among married women aged 15–49 in 2000 was 6.3%, which was lower than most countries in Eastern Africa. The CPR in Ethiopia subsequently increased to 13.9% in 2005 to 27.3% in 2011 and to 35.3 in 2016 (EDHS 2016). The strong commitments from the Ethiopian government to provide reproductive health service and family planning could certainly have played an important role in improvements in female LE since 2005.

Despite the increases in LE following a decline in mortality from respiratory tract infections (RTIs) for both males and female, mortality from RTIs was the main cause of sex differences in LE during all the study periods. Previous studies have shown that females are more commonly affected by infections of the upper respiratory tract, while males are more commonly affected by lower RTIs, and the course of most RTIs is more severe in males than in females, leading to higher mortality in males [30]. Prior systematic reviews and empirical studies have also shown the incidence of admissions for lower RTIs is higher for boys than for girls for all regions globally, including Ethiopia [31–34]. Although the higher risk could be attributable to biological reasons, such as the smaller airway size in young boys than in young girls [35], Nair et al. pointed out that the substantial sex differences in India, Pakistan, and Bangladesh probably show the importance of cultural factors, such as preference in seeking medical care for boys [31]. Savitha and Gopalakrishnan demonstrated that the higher number of RTIs by boys in India is probably attributed to the tendency of male children to play more outside the home, which exposes them to infected aerosols from the surrounding outdoor environment, when compared to female children [36]. On the other hand, an estimated 95% of the population of Ethiopia uses traditional biomass fuels, such as wood, dung, charcoal, or crop residues, to meet household energy needs, resulting in high indoor air pollution [37]. A study on the 24-h concentration of NO (2) in rural setting of Ethiopia showed level of 97 μg/m^3^, which is higher than double the currently proposed annual mean of WHO air quality guidelines [38, 39]. Furthermore, evidence about close quantitative relationships between exposure to high concentrations of particulate matters and increased mortality from respiratory and cardiovascular diseases may indicate that Ethiopian females mainly responsible for household food cooking might be more exposed to indoor air pollution than males, which in tern predisposes them to a higher incidence of infections of the upper respiratory tract. In this context, to what extent and in which direction sex differences cause higher mortality from respiratory infections in males in Ethiopia is uncertain and therefore these differences deserve further research and health policy attention to reduce inequity in health between men and women.

Despite the smaller contributions of injuries to LE improvements for both males and females, except during 2000–2005, mortality from injuries persistently explain the major sex differences in LE across the study periods. While the higher figure in 2000 might be attributed to the impacts of the Eritrean-Ethiopian war, which claimed the lives of more males than females [40], the sustained effects on gains in LE for women from injuries indicates that men are more exposed to interpersonal violence and traffic injuries. Evidence suggests that most injuries in Ethiopia are road traffic injuries [41–45] and most involve young males [43, 46]. A study on the incidence of interpersonal violence in Northwest Ethiopia showed that the incidence of interpersonal violence-related injuries was 28.5%, mainly affecting the 20–29 age group [47]. Alcohol use was the most significant factor associated with interpersonal violence-related injury and male drivers also tend to have lower attention, patience and risk perception than females [48]. According to the latest WHO data published in 2018, road traffic accidents deaths in Ethiopia accounted for 4.81% of total deaths, and the age-adjusted mortality rate is 36.78 per 100,000 of population which ranks Ethiopia 24th in the world [49]. Road injuries, falls, self-harm and interpersonal violence were the leading causes of mortality from injuries occurring in 2017, among which deaths of males, children under 5 years, and people aged 15–24 had the highest share [50].

Our results also shows that HIV/AIDS and STIs had a negative contribution to gains in LE for both males and females from 1995 to 2000. This coincides with the peak in HIV/AIDS incidence rate in Ethiopia in 1995 [51, 52]. After 1995, the incidence declined annually by 6.3%, reaching a 77% reduction between 1990 and 2016 [52]. According to Deribew et.al., the age- standardised incidence rate declined from 178 to 40 per 100,000 population between 1990 and 2016 [52]. The results in our analysis show that after 2000, where national public health care and treatment improved, HIV/AIDS contributed positively to the improvement in LE both for males and females. On the other hand, the contributions of HIV/AIDS to the sex differences in LE in Ethiopia were negative from 1995 to 2015, which indicates the higher morbidity and mortality among women than men in Ethiopia [53]. Recent studies have also argued that armed-conflicts or public health crises, such COVID-19, prevent women in need of reproductive health care from having necessary and even life-saving care and attention, mainly, due to the shutdown of routine health services or fear of infection, which prevent them from going to health facilities [54–57]. In the face of frequent civil unrest and the recent COVID-19 in Ethiopia, the Ethiopian government should strive to ensure access to sexual and reproductive health services for women.

In terms of the recent increasing discrepancy in sex differences in LE since 2005, the results on cause-specific mortality are in line with what previous studies have found; namely, that most of the increases in sex differences in LE are due to increases in male mortality rates from NCDs relative to those of females [3, 6, 58]. Our decomposition results showed that the increasing sex differences in LE since 2005 were largely attributable to the growing male disadvantage in mortality rates from NCDs. In other words, male excess mortality increased during the long-term demographic/epidemiolocal transition in which infectious disease mortality was replaced by chronic disease mortality among adults. Previous studies have demonstrated that cardiovascular diseases are responsible for 80% of the increases in sex differences [58]. Our results show that male mortality from cardiovascular diseases started increasing sex difference in LE from 2015. This reflects changes in smoking and other behavioural or lifestyle factors, which may have affected men more than women as the age-specific contribution to sex differences in LE by age groups above the age of 40 years consistently increased since 2010 in Ethiopia. This result also means that elderly Ethiopian women have a better caring capacity and men are less capable of coping in patriarchal societies such as Ethiopia. This requires further investigation and approaches to narrow the sex differences in LE in Ethiopia where there is a weak social welfare and security system.

Our study has several limitations. First, our results rely on the quality of the estimates of the numbers of deaths from the GBD 2017. While the GBD estimates are considered reliable and robust, they are necessarily limited by the quality of the available data, evidenced by large 95% uncertainty range of ASMRs. Although this study attempted to address this uncertainty by providing uncertainty ranges of LE and ASMRs for males and females, this study was unable to provide confidence intervals for age- and cause- specific contributions to sex differences in LE. Therefore, our result may have either under- or overestimated the true contribution of a specific cause of deaths. Further study is required to estimate confidence intervals of age- and cause- specific contributions to sex differences in LE over several decades. Secondly, we particularly focused on the top seven causes of death, and thus the emerging causes of death from the ‘other CDs’ and ‘other NCDs’ categories in Ethiopia may have been missed. Thirdly, this study explored age-and cause- specific contributions to LE for both males and females and sex differences in LE between 1995 and 2015 in five-year intervals. More granular patterns in LE and sex differences in LE could be obtained by exploring each single year. Lastly, this study only presented a national picture of LE and sex differences in LE. Nevertheless, in Ethiopia, as well as in many sub-Saharan African countries, there are considerable and growing regional and community variations in socioeconomic and security contexts and thus regional and community lens within a sub-Saharan African country are very important for understanding the patterns of LE and sex differences in LE in sub-Saharan Africa. Further research is needed to investigate such variations between regions and community in the increase in sex differences in LE in Ethiopia. However, this approach is challenging since many sub-Saharan African countries lag far behind the rest of the world in civil registration and vital statistics (CRVS) systems. Comparative studies on age- and cause- specific contributions to sex differences in LE between different regions within sub-Saharan Africa should be carried out with advanced CRVS systems, since such studies can contribute to evidence-based and context-specific health policies in pursuit of improved health outcomes, including sustainable improvements in LE for both men and women [12, 59].

## Conclusions

Despite the increments in LE for both Ethiopian men and women for the last three decades, sex differences in LE have also increased recently. While higher male mortality from injuries and respiratory infections were the consistent causes of sex differences in LE in Ethiopia, increasing male morality from NCDs was the main cause of the more recent increase in sex differences in LE in Ethiopia. However, unlike the higher exposure of males to death from injuries due to road traffic injuries or interpersonal violence, to what extent sex differences cause higher male mortality from respiratory infection diseases is unclear. Similarly, despite Ethiopia’s weak social security system, an explanation of the increased sex differences in LE after the age of 40 years due to either longer female LE or reduced male LE should be further investigated.

## Supplementary Information

**Additional file 1.**

## Data Availability

All data used in this study is publicly available through websites the Global Health Data Exchange (http://ghdx.healthdata.org).

## Abbreviations

ASMR: Age-standardised Mortality Rate
CD: Communicable diseases
CMF: Comparative Mortality Figure
CMNND: Communicable, maternal, neonatal and nutritional disease
CVD: Cardiovascular disease
GBD: Global Burden Disease
IHME: The Institute for Health Metrics and Evaluation
LE: Life Expectancy
NCDs: Non-communicable diseases
