# Supplementary material for: The triple burden of communicable and non-communicable diseases and injuries on sex differences in life expectancy in Ethiopia
Source: Int J Equity Health. 2021 Aug 3;20:180. doi: 10.1186/s12939-021-01516-0 (PMC8330193; doi:10.1186/s12939-021-01516-0)
Supplement: Supplementary file 1 — Additional file 1. [file 12939_2021_1516_MOESM1_ESM.pdf]

Supplementary Material: **Different values verse rate ratio of Life expectancy (LE) between males and females**

We have explored sex differences in LE for five years (1995, 2000, 2005, 2010, 2015) in both absolute and relative measures by overall LE and years. (Figures 1 and 2). Results from absolute and relative measures were quite similar as absolute versus relative measures of sex differences in LE were in close agreement (figure 3)

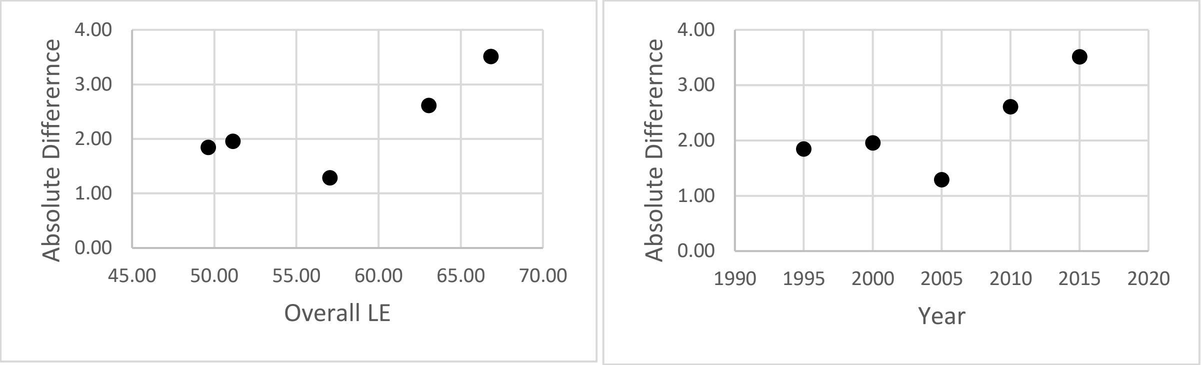

(A) (B)

Figure 1. Life expectancy(LE) difference between males and females by overall LE(A) and years(B)

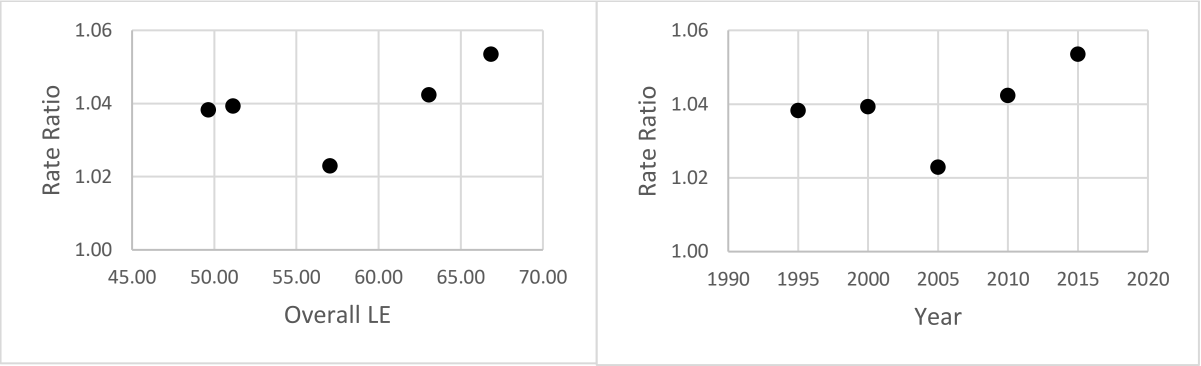

(A) (B)

Figure 2. Rate ratio of Life expectancy(LE) difference between males and females by overall LE(A) and years(B)

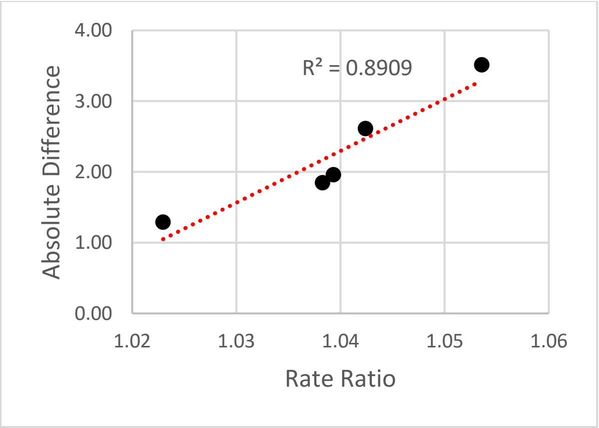

Figure 3. Different values verse rate ratio of Life expectancy(LE) between males and females
